# Maternal high-fat diet programs white and brown adipose tissue lipidome and transcriptome in offspring in a sex- and tissue-dependent manner in mice

**DOI:** 10.1038/s41366-021-01060-5

**Published:** 2022-01-07

**Authors:** Christina Savva, Luisa A. Helguero, Marcela González-Granillo, Tânia Melo, Daniela Couto, Byambajav Buyandelger, Sonja Gustafsson, Jianping Liu, Maria Rosário Domingues, Xidan Li, Marion Korach-André

**Affiliations:** 1 Karolinska Institute/AstraZeneca Integrated Cardio Metabolic Center (ICMC), Huddinge, Sweden; 2 Department of Medicine, Metabolism Unit, Karolinska University Hospital Huddinge, Stockholm, Sweden; 3 Clinical Department of Endocrinology, Metabolism and Diabetes, Karolinska University Hospital Huddinge, Stockholm, Sweden; 4 Department of Medical Sciences, Institute of Biomedicine, University of Aveiro, Aveiro, Portugal; 5 Mass Spectrometry Centre, Department of Chemistry, University of Aveiro, Aveiro, Portugal; 6 CESAM, Centre for Environmental and Marine Studies, Department of Chemistry, University of Aveiro, Aveiro, Portugal

**Keywords:** Post-translational modifications, Fat metabolism

## Abstract

**Objective:**

The prevalence of overweight and obesity among children has drastically increased during the last decades and maternal obesity has been demonstrated as one of the ultimate factors. Nutrition-stimulated transgenerational regulation of key metabolic genes is fundamental to the developmental origins of the metabolic syndrome. Fetal nutrition may differently influence female and male offspring.

**Methods:**

Mice dam were fed either a control diet or a high-fat diet (HFD) for 6-week prior mating and continued their respective diet during gestation and lactation. At weaning, female and male offspring were fed the HFD until sacrifice. White (WAT) and brown (BAT) adipose tissues were investigated in vivo by nuclear magnetic resonance at two different timepoints in life (midterm and endterm) and tissues were collected at endterm for lipidomic analysis and RNA sequencing. We explored the sex-dependent metabolic adaptation and gene programming changes by maternal HFD in visceral AT (VAT), subcutaneous AT (SAT) and BAT of offspring.

**Results:**

We show that the triglyceride profile varies between adipose depots, sexes and maternal diet. In female offspring, maternal HFD remodels the triglycerides profile in SAT and BAT, and increases thermogenesis and cell differentiation in BAT, which may prevent metabolic complication later in life. Male offspring exhibit whitening of BAT and hyperplasia in VAT when born from high-fat mothers, with impaired metabolic profile. Maternal HFD differentially programs gene expression in WAT and BAT of female and male offspring.

**Conclusion:**

Maternal HFD modulates metabolic profile in offspring in a sex-dependent manner. A sex- and maternal diet-dependent gene programming exists in VAT, SAT, and BAT which may be key player in the sexual dimorphism in the metabolic adaptation later in life.

## Introduction

The drastic increase in consumption of high caloric diets worldwide with high levels of modified fat by the food industry associated with a sedentary lifestyle, has dramatically challenged humans’ metabolism. Most importantly, the increased prevalence of obesity and overweight women in reproductive age has urged the need to better understand the impact on the fetus health [1]. Recently, several studies have demonstrated the noteworthy sensitivity of the offspring to nutritional environment during the prenatal, neonatal and postnatal periods, which facilitates the development of metabolic complications in adulthood [2–4]. The intrauterine programming of obesity in offspring adulthood relies on genetic regulation as a key mechanism [5]. Genetic modifications by maternal high-fat diet (moHF) lead to a cyclical transgenerational transmission that soon may become a heavy burden worldwide. Moreover, sex-dependent metabolic adaptation to moHF has been recently described by our group and others, but the underlying mechanism remains to be elucidated [4, 6, 7].

Adipose tissue (AT) is a complex and highly metabolically active organ essential for metabolic homeostasis [8]. In mammalians, white and brown adipocytes tune energy balance according to the calorie intake and the energy expended. The development of AT occurs at an early stage, during prenatal and postnatal periods [9], hence moHF may have an impact on programming offspring AT function [10].

In the current study, we first explored how moHF prior and throughout pregnancy and lactation can predispose white and brown AT in offspring fed with the HFD (i.e., obese offspring) to metabolic dysfunctions. This was achieved by (1) characterizing in vivo the metabolic response to moHF in offspring at two different timepoints and (2) exploring the transcriptome and lipidome of visceral (VAT), subcutaneous (SAT), and brown (BAT) AT. Second, we focused on the biological and transcriptional sex-specific response to moHF. Our results showed that moHF does not affect global adiposity in obese offspring but remodeled triglycerides (TG) in AT in a sex-dependent manner. Moreover, we observed sex- and AT-dependent gene regulation by moHF in offspring which may balance AT lipidome and physiology and contribute to the sex-dependent metabolic adaptations in adulthood.

## Results

### Maternal HFD alters adipocyte morphology and adipose transcriptional activity in offspring in a sex-dependent manner

**Fig. 1:**
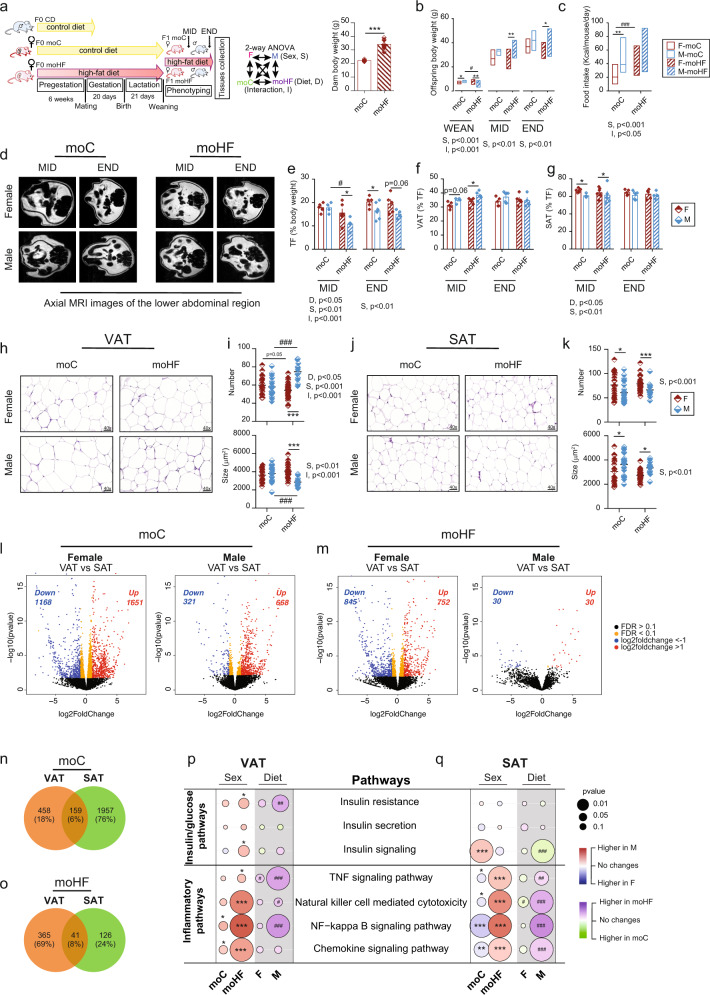
Sex and maternal diet effects on white adipose physiology and biology in male and female offspring. **a** Experimental setting of the study, two-way ANOVA statistical comparisons with sex and maternal diet as two variables, and pre-gestational body weight of dam-F0 in CD group (moC, *n* = 6) (open bar) and HFD group (moHF, *n* = 6) (stripped bar). **b** Body weight of female and male offspring at weaning, midterm (MID) and endterm (END) (F-moC, *n* = 10, M-moC, *n* = 11, F-moHF, *n* = 11 and M-moHF, *n* = 12). **c** Average food intake (Kcal/day/mouse). **d** Representative axial MRI images of the lower abdominal region in female and male offspring (F-moC, *n* = 5, M-moC, *n* = 7, F-moHF, *n* = 6 and M-moHF, *n* = 5). **e** Total fat (% body weight), **f** visceral fat (VAT, % total fat), and **g** subcutaneous fat (SAT, % total fat). Representative images of H&E staining in **h** VAT (F-moC, *n* = 4, M-moC, *n* = 4, F-moHF, *n* = 6 and M-moHF, *n* = 2) and **j** SAT (F-moC, *n* = 5, M-moC, *n* = 5, F-moHF, *n* = 6 and M-moHF, *n* = 3) from moC and moHF offspring. Adipocyte number (hyperplasia) and size (hypertrophy) quantification in **i** VAT and **k** SAT. Volcano plots displaying differently expressed genes (DEG) between VAT and SAT in **l** moC (*n* = 5/sex) and in **m** moHF (females, *n* = 6 and males, *n* = 3). Venn diagram of DEG between sexes in **n** moC and **o** moHF VAT and SAT. Significantly (FDR < 0.1) upregulated (red dots) and downregulated (blue dots) expressed transcripts over log2foldchange >1 and <−1. Orange dots indicate the significantly regulated genes (FDR < 0.1) and the black dots the not significant genes. Bubble charts showing the sex- and maternal diet effects on the selected insulin and inflammatory KEGG pathways in **p** VAT and **q** SAT (F-moC, *n* = 5, M-moC, *n* = 5, F-moHF, *n* = 6 and M-moHF, *n* = 3). The white background represents the sex comparison and the gray background the mother diet comparison. The color of the bubbles indicates the maximum estimate score (MES) between the groups where red indicates upregulation in males and blue upregulation in females (sex effect). The green color indicates upregulation in moC and purple indicates upregulation in moHF (maternal diet effect). The size of the bubble indicates the level of significance. F females and M males. Two-way ANOVA (sex (S), mother diet (D), interaction (I) between sex and diet) followed by a Tukey’s multiple comparisons test when significant (*p* < 0.05) in **b**, **c**, **e**–**g**, **i** and **k**. Benjamini–Hochberg correction with FDR < 0.1 and *p* < 0.05 was used for **l**–**q**. *males vs females and ^#^moHF vs moC (*p* < 0.05); **p < 0.01; ***p < 0.001. RPKM reads per kilo base per million mapped reads.

At the age of 5 weeks, C57bl/6 dams were fed with the control diet (CD) or the high-fat diet (HFD) for 6 weeks prior mating, during pregnancy and lactation. F0 sires were fed with the CD. After weaning, all F1 offspring were fed with the HFD. Prior mating, the HFD-dam (moHF) weighted significantly more than CD-dam (moC) (Fig. 1a). At weaning, offspring body weight was sex- and maternal diet-dependent. At midterm (MID) and endterm (END) males from moHF (M-moHF) weighted more than females from moHF (F-moHF) (Fig. 1b). The food intake was higher in M-moC than in F-moC; moHF increased the food intake in females only (Fig. 1c). We used in vivo magnetic resonance imaging to measure total adiposity in offspring in short (MID) or/and long (END) term. At MID, M-moHF accumulated less fat than M-moC and females had more fat than males at END (Fig. 1d, e). Only at MID, females had lower proportion of VAT and higher of SAT than males (Fig. 1f, g).

Changes in AT morphology can trigger tissue dysfunction [8]. H&E staining revealed that M-moHF showed hyperplasia and reduced hypertrophy compared to M-moC and F-moHF (Fig. 1h, i). In SAT, males showed hypertrophy and reduced hyperplasia compared to females (Fig. 1j, k). Analysis from smart-Seq2 revealed that the number of differentially expressed gene (DEG) between VAT and SAT in M-moC was smaller than in F-moC and almost negligible in moHF (Fig. 1l, m). We then explored the DEG between sexes. In moC, most of the DEG between sexes were in SAT (Fig. 1n). Surprisingly, moHF considerably reduced the number of DEG only in SAT (Fig. 1o). Collectively, our results reveal that moHF affects the offspring transcriptome in a sex- and adipose depot-specific manner.

### Maternal HFD modulates offspring susceptibility to insulin resistance and inflammation in a sex-dependent manner

To assess biological parameters indicative of metabolic dysfunctions, we measured fasting glucose and insulin levels (Table 1). At MID, glucose was similar between sexes in moC but higher in M-moHF than in F-moHF. Insulin level and homeostatic model assessment (Homa) index were higher in males than in females but was increased by moHF in females only. Matsuda index, a marker of whole-body insulin sensitivity, was higher in females than males; moHF reduced it in females at MID. At END, the ratio AUCins:AUCglc, a marker of β-cell function, was impaired in M-moC compared to F-moC, but moHF impaired females at END (Table 1).

**Table 1:** Plasma parameters in female (F) and male (M) offspring.

	MIDTERM				ENDTERM			
Diet	moC		moHF		moC		moHF	
Sex	F	M	F	M	F	M	F	M
Glucose (mM)	8.9 ± 0.5	10.8 ± 0.4	7.6 ± 0.5	13.0 ± 01.2***	8.7 ± 0.1	11.7 ± 0.6**	7.5 ± 0.5	12.4 ± 1.2***
Insulin (ng/ml)	0.69 ± 0.08	2.24 ± 0.25***	1.40 ± 0.17^#^	3.89 ± 1.22**	0.61 ± 0.10	5.43 ± 1.08**	1.37 ± 0.20^##^	7.27 ± 1.17***
Homa index	0.27 ± 0.03	1.07 ± 0.12**	0.47 ± 0.07^#^	2.81 ± 0.87***^#^	0.24 ± 0.04	2.89 ± 0.62**	0.48 ± 0.10^#^	3.98 ± 0.64***
Matsuda index	1412 ± 143	387 ± 45***	806 ± 84^##^	289 ± 80***	1644 ± 285	265 ± 97***	994 ± 169	149 ± 48***
AUCins:AUCglc	0.06 ± 0.01	0.17 ± 0.02***	0.15 ± 0.02	0.17 ± 0.02	0.06 ± 0.01	0.33 ± 0.06**	0.20 ± 0.03^##^	0.36 ± 0.07

Plasma levels and markers of insulin sensitivity in female and male moC and moHF offspring. For glucose mice were fasted for 6 h and for insulin mice were fasted for 4 h prior to the blood sampling from the tail. Data are presented as mean ± SEM. For F-moC, *n* = 7; for M-moC, *n* = 8; for F-moHF, *n* = 7; for M-moHF, *n* = 8.

*F* female, *M* male, *Homa* homeostatic model assessment.

*M vs F and ^#^moHF vs moC. ^#^*p* < 0.05; ** or ^##^*p* < 0.01; ***p < 0.001.

Obesity alters AT function and activates basal systemic inflammation, which in turn promotes the development of insulin resistance. Pathway analysis and chord plot was performed to show a detailed relationship between the regulation of the genes (left semicircle) between sexes (1-sex differences in moC and 2-sex differences in moHF) and between maternal diet (3-moHF effect in females and 4-moHF effect in males) and their enriched pathways (right semicircle). Males showed induced insulin resistance and reduced insulin signaling pathway activity with moHF, in VAT and SAT respectively. F-moHF showed increased expression of insulin signaling genes in VAT (*Pck1, Slc27a1*, and *Insr*). Inflammatory pathways were upregulated in VAT (higher expression of *Ccl9*, *Dock2*, *Ifngr1, and Tnfrsf1a*), and downregulated in SAT in M-moC compared to F-moC, but moHF increased inflammatory pathways in males only (Figs. 1p, q and S1a, b and S2a, b).

### Maternal HFD modulates triglycerides composition in visceral and subcutaneous adipose tissue in a sex-dependent manner

**Fig. 2:**
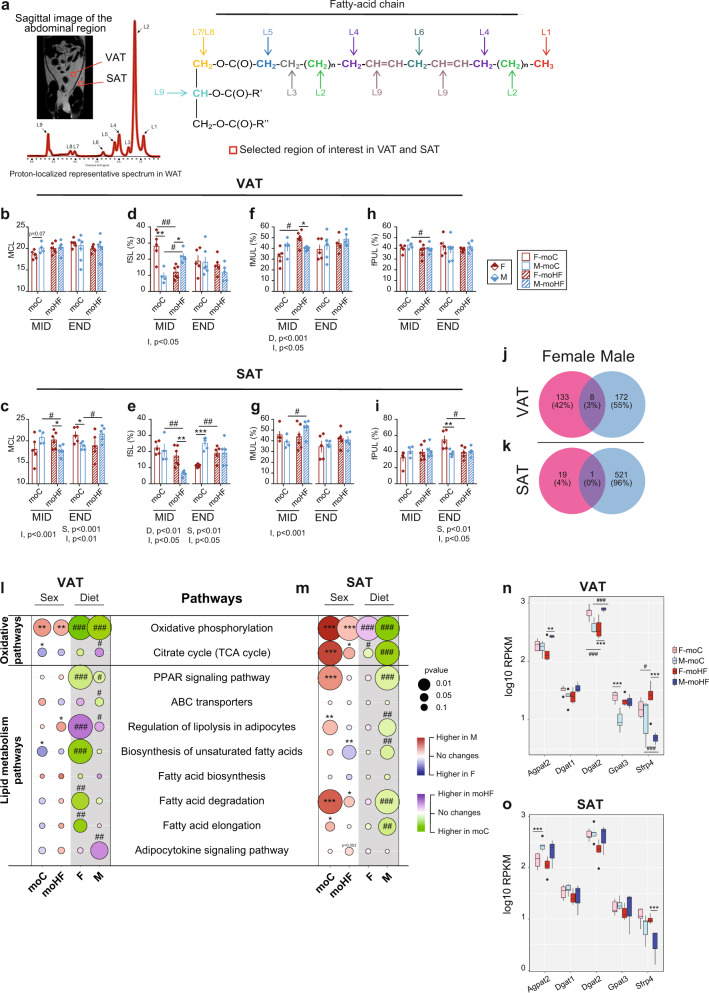
Sex and maternal diet modulate triglycerides composition in white adipose tissue of offspring. **a** Sagittal image of the whole-body fat and one representative ^1^H-localized spectrum for in vivo quantification of fatty acids composition of the triglyceride molecule in offspring’s WAT (F-moC, *n* = 5, M-moC, *n* = 7, F-moHF, *n* = 6 and M-moHF, *n* = 5). Medium chain length (MCL) in **b** VAT and **c** SAT. Fraction of saturated lipids (fSL) in **d** VAT and in **e** SAT; Fraction of monounsaturated lipids (fMUL) in **f** VAT and in **g** SAT; Fraction of polyunsaturated lipids (fPUL) in **h** VAT and in **i** SAT. Venn diagram displaying the DEG by moHF in female and male offspring in **j** VAT and in **k** SAT. Bubble charts showing the sex- and maternal diet effects on the oxidative and lipid metabolism KEGG pathways in **l** VAT and in **m** SAT. Boxplot of the selected genes of the lipid metabolism in **n** VAT and **o** SAT (log10(RPKM)). For RNA sequencing analysis F-moC, *n* = 5, M-moC, *n* = 5, F-moHF, *n* = 6 and M-moHF, *n* = 3. The white background represents the sex comparison and the gray background the mother diet comparison. The color of the bubbles indicates the maximum estimate score (MES) between the groups where red indicates upregulation in males and blue upregulation in females (sex effect). The green color indicates upregulation in moC and purple indicates upregulation in moHF (maternal diet effect). The size of the bubble indicates the level of significance. F females and M males. Two-way ANOVA (sex (S), mother diet (D), interaction (I) between sex and diet) followed by a Tukey’s multiple comparisons test when significant (*p* < 0.05) in **b**–**i**. Benjamini–Hochberg correction with FDR < 0.1 and *p* < 0.05 was used for **j**–**o**. *males vs females and ^#^moC vs moHF (*p* < 0.05), **^, ##^*p* < 0.01; ***^,###^*p* < 0.001. RPKM reads per kilo base per million mapped reads.

Sex-specific TG composition in fat depot could trigger the sex-dependent differences in obesity. Therefore, we performed in vivo proton magnetic resonance spectroscopy (^1^H-MRS) in WAT of offspring (Fig. 2a).

In VAT, no differences of mean chain length (MCL) were observed between all groups and at both timepoints, despite higher expression of *Elovl*3 in M-moC and *Elovl6* in F-moC (Figs. 2b and S3a). In contrast, in SAT at MID, moHF reduced MCL in males to a lower level than in females. At END, F-moC had longer MCL than M-moC, and higher expression of *Elovl6* and *Elovl7*; moHF increased MCL and *Elovl7* expression in males (Figs. 2c and S3b). In VAT, at MID the fraction of saturated lipids (fSL) was higher in F-moC than in M-moC; moHF respectively decreased and increased the fSL in females and males (Fig. 2d). In SAT at MID, M-moHF had lower fSL than F-moHF and M-moC (Fig. 2e). At END, the fSL was higher in M-moC than in F-moC, with lower expression of *Elovl6* (Figs. 2e and S3b). In VAT at MID, F-moHF increased the fraction of monounsaturated lipid (fMUL) to a higher level than M-moHF (Fig. 2f). In SAT, fMUL was increased by moHF in males at MID (Fig. 2g). In VAT, moHF reduced the fraction of polyunsaturated lipid (fPUL) in males at MID (Fig. 2h). In SAT, the fPUL was higher in F-moC than in M-moC but moHF reduced the fPUL in females at END (Fig. 2i). In sum, moHF provokes TG remodeling in VAT and SAT at MID in a sex-dependent manner, but at END, TG profile is modulated only in SAT.

### Maternal HFD modulates the white adipose transcriptome differently between sexes and between adipose depots

PCA plots showed a distinct separation of the two fat pads demonstrating a clear heterogeneity in the transcriptome characteristics (Fig. S4a). Sexes separately clustered in VAT and SAT, with an effect of moHF in males (Fig. S4b, c). We then inspected the number of DEG by moHF in VAT and SAT. Interestingly, most of the DEG by moHF were sex-specific; moHF modulated the transcriptional activity in male’s SAT while 50% of DEG were found in VAT in both sexes (Fig. 2j, k).

Understanding the adipose specific TG synthesis and hydrolysis/oxidation is critical to better predict the development of obesity. In VAT and SAT, the oxidative phosphorylation pathway and oxidative gene expression (*Tcirg1*, *Atp6v0e*, and *Atp6v0d2*) were upregulated in M-moC compared to F-moC (Figs. 2l, m and S1a and S2a). In VAT, moHF upregulated lipolysis and downregulated PPAR signaling and FA synthesis pathways in females (Fig. 2l). In SAT, moHF upregulated the oxidative phosphorylation pathway but downregulated the citrate cycle pathway in females and downregulated all oxidative pathways in males (Fig. 2m). In SAT, lipid metabolism pathways were upregulated in M-moC compared to F-moC together with *Mgll, Pnpla2, Cpt2*, and *Me1* genes (Figs. 2m and S2a). moHF repressed most of the lipid pathways in males. In VAT, moHF reduced and induced the expression of the *Dgat2* gene (involved in TG re-esterification) in females and males respectively (Fig. 2n). Inversely, moHF induced and reduced *Sfrp4* expression (involved in adipogenesis) in females and males respectively, with higher expression level in F-moHF than M-moHF in both fat pads (Fig. 2n, o). In sum, we reveal that moHF alters the TG profile in AT in a sex-dependent manner, likely due to sex-specific reprogramming of genes involved in the elongation and desaturation of the TG.

### Maternal HFD alters triglycerides composition and gene expression in the brown adipose tissue of female offspring

**Fig. 3:**
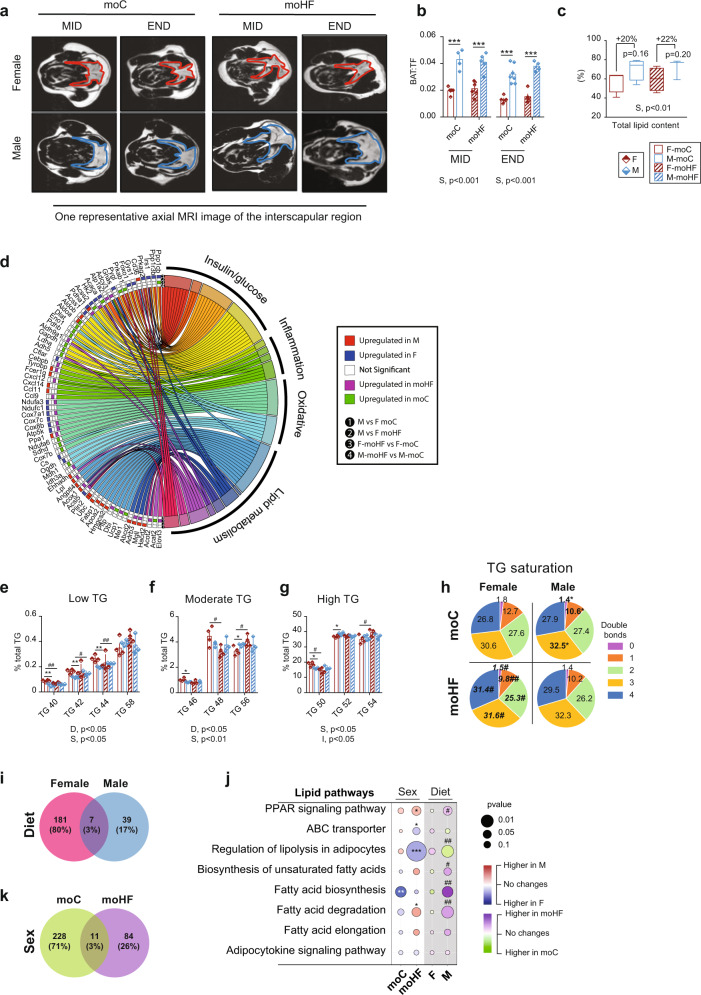
Brown adipose tissue offspring metabolism is altered by maternal obesity. **a** MRI images of the interscapular brown adipose tissue (BAT) in female and male offspring. **b** Quantification of BAT on total fat (BAT:TF) ratio based on MRI images (F-moC, *n* = 5, M-moC, *n* = 7, F-moHF, *n* = 6 and M-moHF, *n* = 5). **c** Total lipid content in BAT (in percent of the total mass) (*n* = 4/group). **d** Chord plot displaying the DEG clustered into selected pathways in BAT. Female vs male (sex comparison) in (1) moC and (2) moHF, and moC vs moHF (diet comparison) in (3) females and (4) males. Low abundant **e** Moderate abundant **f** and High abundant **g** TG classes (*n* = 4/group). **h** TG saturation profile. Venn diagram showing the relationship between the DEG by **i** moHF in females and males and by **k** sexes in moC and moHF. **j** Bubble chart showing the sex- and maternal diet-dependent regulation of selected KEGG lipid metabolic pathways in BAT. For RNA sequencing analysis F-moC, *n* = 5, M-moC, *n* = 5, F-moHF, *n* = 6 and M-moHF, *n* = 3. The white background represents the sex comparison and the gray background the mother diet comparison. The color of the bubbles indicates the maximum estimate score (MES) between the groups where red indicates upregulation in males and blue upregulation in females (sex effect). The green color indicates upregulation in moC and purple indicates upregulation in moHF (maternal diet effect). The size of the bubble indicates the level of significance. F females and M males. Two-way ANOVA (sex (S), mother diet (D), interaction (I) between sex and diet) followed by a Tukey’s multiple comparisons test when significant (*p* < 0.05) in **b**, **c**, **e**–**g**. Differences between two groups in **h** (sexes, F vs M; maternal diet, moC vs moHF) were determined by unpaired *t*-test corrected for multiple comparisons using the Holm–Sidak method, with alpha = 5.000%. *males vs females and ^#^moHF vs moC (*p* < 0.05); **^,##^*p* < 0.01; ***^,###^*p* < 0.001. Benjamini–Hochberg correction with FDR < 0.1 and *p* < 0.05 was used for **d**, **i**–**k**.

Adiposity is balanced depending on energy intake and energy expenditure. Energy intake was modified in F-moHF with unchanged global adiposity. BAT is distinguished from WAT by a high level of mitochondria containing UCP1, a unique protein disconnecting the oxidative phosphorylation from ATP synthesis and inducing thermogenesis [11]. When activated, thermogenesis has a major impact on local and systemic energy balance. MRI revealed that males showed twice as big BAT than females, containing 20% more TG with higher expression of *Cd36*, *Cpt1*, *Plin2*, and *Fabp1* (Fig. 3a–d). In obesity, modulation of BAT activity and TG profile has been associated to insulin resistance [12] and to be sex-specific [13]. We performed a lipidomic analysis of harvested BAT and quantified ten TG classes. TG classes were classified into low, moderate, and high abundance. TG40-TG50 were more abundant and TG52 and TG56 less abundant in F-moC than M-moC (Fig. 3e–g). TG50 abundance was decreased and TG54 increased in F-moHF only (Fig. 3g). These results show that in addition to sex differences, moHF selectively modulates TG classes in BAT.

We next dissected the 54 TG species detected (Fig. S5a) and found that 17 of 54 are characterized with sex-differences in moC but only 2 of 54 in moHF. These changes were attributed to a remodeling of TG species with moHF in females (15 of 54); as favored by increased expression of *Lpl*, *Pltp, Dbi*, and *Elovl3* genes by moHF (Fig. 3d). Metabolic diseases are associated with the degree of hydrogen atom bond saturation within the FA contained into TG molecules [14]. The saturation profile was highly sex dependent in moC but, moHF altered the saturation level in females only (Fig. 3h). FA as signaling molecules may contribute to metabolic dysfunction in obesity. Ten FA species were detected, C18:3ω6 relative level was lower in M-moC than F-moC, and two FA were modified by moHF in both sexes (Fig.  S5b). Total ω3-FA and ω6-FA levels were higher in females than in males in moHF and moC respectively. The ω6:ω3 ratio was increased by moHF in males, indicator of impaired metabolic profile [15] (Fig. S5c).

PCA plots of the RNA-seq data showed a distinct separation of the WAT and BAT with clear heterogeneity in the transcriptome characteristics between them (Fig. S4a). In BAT, PCA clustered females from moC and moHF separately, while males were more spread (Fig. S4d). We then inspected the number of DEG by moHF in females and males and the interrelation between sexes. Most of the DEG were found in females (80%) with only seven DEG shared between sexes (Fig. 3i). However, analysis revealed that moHF modulated lipid pathway activity in males only (Fig. 3j). Interestingly, 71% of the DEG between sexes were found in moC, while only 26% in moHF group (Fig. 3k).

### Maternal HFD oppositely regulates inflammation and oxidative phosphorylation pathways between sexes in offspring’s BAT

**Fig. 4:**
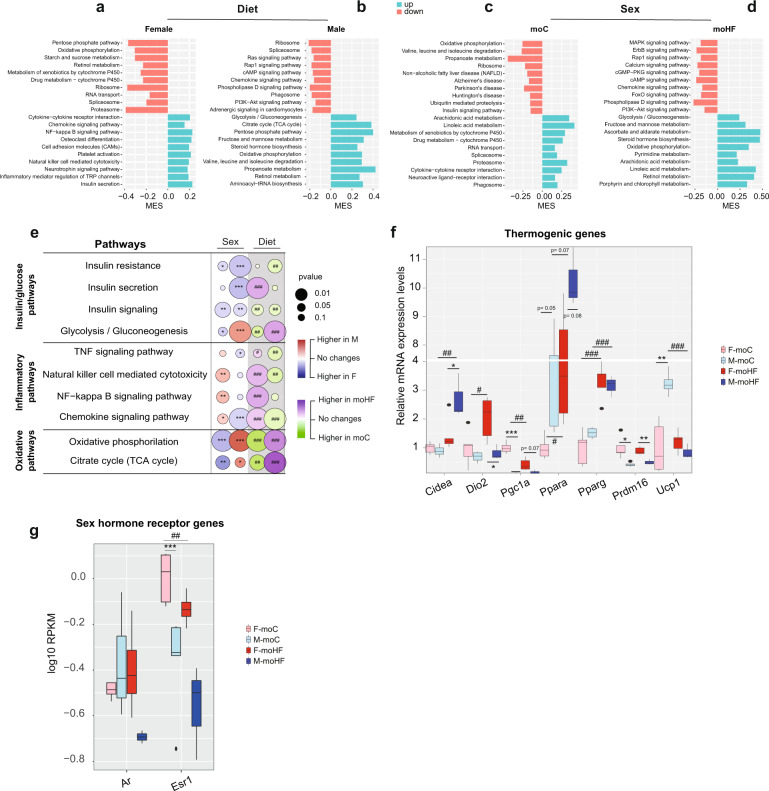
Maternal HFD reprograms metabolic pathways and gene expression in offspring brown adipose tissue in a sex-specific manner. The top 10 significantly upregulated and downregulated KEGG pathways between maternal diets in **a** female and in **b** male offspring and between sexes in **c** moC and in **d** moHF in BAT. **e** Bubble chart showing the sex- and maternal diet effect on selected insulin/glucose, inflammatory and oxidative phosphorylation KEGG pathways in BAT. **f** Relative mRNA expression levels of the thermogenic genes in BAT measured by qPCR. **g** Boxplot of the sex hormone receptor gene expression levels in BAT (log10(RPKM)). For F-moC, *n* = 5, M-moC, *n* = 5, F-moHF, *n* = 6 and M-moHF, *n* = 3. The white background represents the sex comparison and the gray background the mother diet comparison. The color of the bubbles indicates the maximum estimate score (MES) between the groups where red indicates upregulation in males and blue upregulation in females (sex effect). The green color indicates upregulation in moC and purple indicates upregulation in moHF (maternal diet effect). The size of the bubble indicates the level of significance. F females and M males. Benjamini–Hochberg correction with FDR < 0.1 and *p* < 0.05 was used for **a**–**e** and **g**. Differences between two groups in **f** (sexes, F vs M; maternal diet, moC vs moHF) were determined by unpaired *t*-test corrected for multiple comparisons using the Holm–Sidak method, with alpha = 5.000%. *males vs females and ^#^moHF vs moC (P<0.05); **^, ##^*p* < 0.01; ***,^###^*p* < 0.001. RPKM reads per kilo base per million mapped reads.

To further investigate the effect of moHF and sex on the activity of transcriptional pathways in BAT offspring, we extracted the top 10 significantly up- and downregulated KEGG pathways. In females, moHF downregulated metabolic and oxidative phosphorylation processes, while upregulated immune inflammatory processes and insulin secretion (Fig. 4a). In males, moHF downregulated cell proliferation and cell survival processes and upregulated metabolic pathways (Fig. 4b). In moC, we found lower oxidative phosphorylation and catabolic processes activity and higher metabolic and cell signaling activity in males than in females (Fig. 4c). In moHF, males showed lower ErbB and Rap1 signaling pathways and cell survival processes and higher glucose and lipid metabolism pathway activity compared to females (Fig. 4d). Altogether these results indicate that BAT transcription of genes in offspring is sex-specific and that moHF reprograms the BAT transcriptome in offspring in a sex-dependent manner, which may contribute to the sex dependent BAT biology.

Further, we extracted selective KEGG metabolic pathways that are fundamental for the intracellular activity of the brown adipocyte and in turn for the metabolism of the BAT (Fig. 4e). moHF upregulated insulin secretion but downregulated insulin signaling pathway in females and, downregulated all insulin pathways in males (Fig. 4e). Females showed higher expression of the insulin pathways than males and, enrichment of genes clustered in metabolic pathways such as *Irs1*, *Ppp1cb*, *Hk2*, *Foxo1, Acaca*, and *Prka* (Fig. 3d). The glucose pathway was higher expressed in F-moC than M-moC, but moHF down- and upregulated the pathways in females and males, respectively (Fig. 4e). Transcriptional activity of several genes (*Pppc1cb, Prkab1, Pygl, Adcy3, Atp1a2, Acaca, Acss2, Aldoa, Dlat, Eno1, Pdhb, Aldh9a1, Gadph, Ldha*, and *Adh5*) of the insulin and glucose pathways was altered in females by moHF, which reduced the differences between sexes (Fig. 3d). Inflammatory pathways were higher expressed in M-moC than in F-moC, and moHF oppositely regulated them between sexes. moHF induced inflammatory pathways in females, together with induced expression of *Ccl9*, *Cxcl12*, *Tyrobp*, and *Cebpb* genes (Figs. 3d and 4e). The oxidative pathways were oppositely regulated between sexes and in response to moHF (Fig. 4e). Expression level of *Atp5k*, *Cox8b*, *Cox7a1*, and *Ndufa3* genes of the phosphorylation pathways was higher in F-moC than M-moC. However, moHF enhanced their expression levels in males only (Fig. 3d).

BAT has the unique ability to burn TG through the thermogenic pathways. In males, moHF induced expression of *Cidea* and *Pparγ* but decreased the expression of *Ucp1*. In females, moHF induced the expression of *Ucp1*, *Dio2, Pparα*, and *Pparγ*, and decreased the expression of *Pgc1α* (Fig. 4f). Males showed higher expression levels of *Cidea* but lower levels of *Dio2*, *Pgc1α* and *Prdm16* than females in moHF. In female, moHF increased the expression of the β3-adrenergic receptor (*Adrβ3*), a key player in energy consumption and lipolysis (Fig. 3d) [16–18]. BAT expresses the major sex hormone receptors *Ers1* and *Ar*, supporting the hypothesis that differences in sex hormone receptor levels may contribute to the sexual dimorphism in BAT activity. The expression of *Ers1* was higher in F-moC compared to F-moHF and M-moC, but not *Ar* (Fig. 4g). To sum, moHF increases the susceptibility to thermogenesis in female offspring and to inflammation and oxidative phosphorylation in males.

### Sex chromosome-linked genes contribute to the sexual dimorphism in offspring adipose transcriptome

Sex chromosomes are important for the physiological development and influence a plethora of complex attributes. To dissect the contribution of sex chromosome complement in offspring health, we presented the X- and Y-linked DEG between sexes involved in the metabolic and embryonic development pathways.

**Fig. 5:**
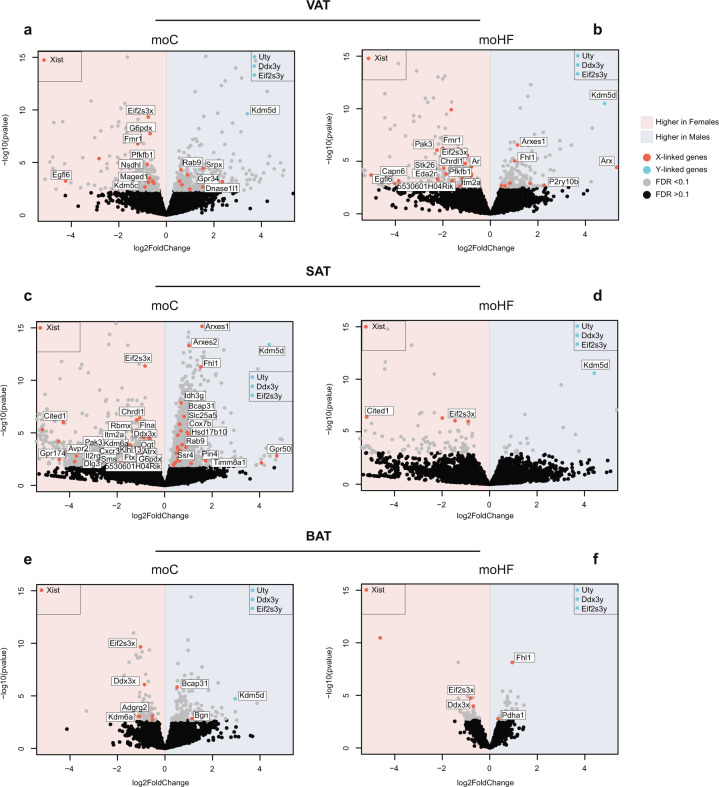
Sex-linked chromosomes contribute to the sexual dimorphism in offspring adipose transcriptome. Volcano plots of the DEG between females and males in **a** moC and **b** moHF in VAT; in **c** moC and in **d** moHF in SAT and in **e** moC and in **f** moHF in BAT with selected genes linked to the X and Y chromosomes involved in metabolism and embryonic development. Genes residing on the X-chromosome are marked in red and those on the Y-chromosome are marked in light blue. The pink background indicates upregulation in females and the blue background indicates upregulation in males. Gray dots indicate significantly deregulated genes (FDR < 0.1) and black dots indicate non-significant genes. Genes that are highly significant with foldchange outside the range of the graph are placed in boxes on the side in the graph. F-moC, *n* = 5, M-moC, *n* = 5, F-moHF, *n* = 6 and M-moHF, *n* = 3.

In VAT, X-linked genes involved in glucose metabolism (*G6pdx*, *Pfkb1*, and *Fmr1*) [19, 20] and for the expansion and differentiation of AT (*Kdm5c*) [21], were induced in F-moC compared to M-moC (Fig. 5a). *Egfl6*, a growth factor upregulated in obesity and involved in AT growth in humans [22], was higher expressed in females than males. The adipogenic gene *Arxes1* [23] was upregulated and *Ar*, involved in fat accumulation [24] was downregulated in M-moHF compared to F-moHF (Fig. 5b). In SAT, we identified several X-linked DEG involved in adipogenesis and beiging processes (*Itm2a*, *Flna, Cited1, Arxes1/2* and *cox7b*) [23, 25–27] (Fig. 5c). However, in moHF the number of X-linked DEG was reduced dramatically (Fig. 5d). In BAT, few X-linked DEG were detected between sexes. In moC, the inflammatory gene *Kdm6a* [28] was upregulated, and *Bgn*, involved in insulin signaling, was downregulated in females. In moHF, *Pdha* mitochondrial gene was upregulated in males (Fig. 5e, f).

**Fig. 6:**
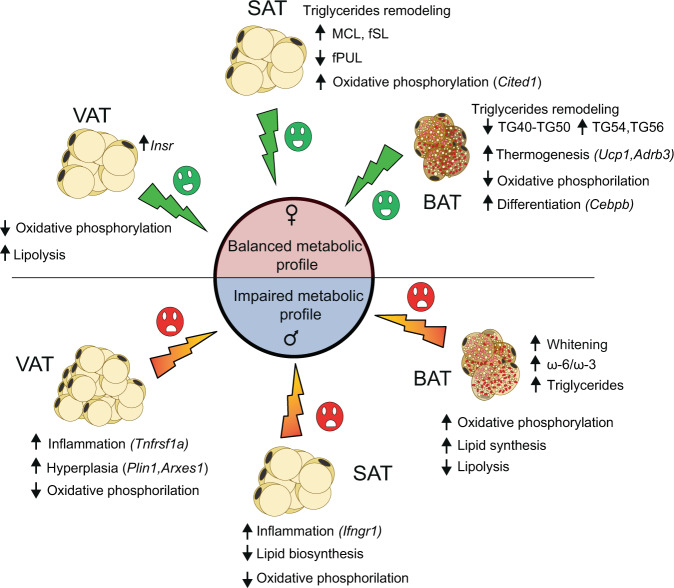
Graphical representation of the main findings in female and male offspring adipose tissue’s metabolic adaptation to moHF. The pink and blue hemispheres represent the effect of moHF in female and male offspring, respectively. The green and the red thunderstruck symbols represent respectively, a positive and a negative signal on the metabolism. The black arrows represent up- or downregulation.

Overall, the results of this study establish that moHF modulates lipid metabolism differently in female and male offspring as summarized in Fig. 6. We demonstrate that in response to moHF, both WAT and BAT undergo to transcriptional, biological and physiological modifications in a sex- and tissue-dependent manner. Female offspring born from obese mothers modulate AT function to maintain metabolic balance, i.e., TG remodeling in SAT and BAT, and increase brown adipocyte differentiation and thermogenesis. Oppositely, male offspring increase hyperplasia in VAT, and induce inflammatory response in both VAT and SAT. In BAT, moHF drives whitening and increases ω6/ω3 ratio and inflammation which in turn impairs metabolic profile.

## Discussion

The recent discovery of the changes in the transcriptional and posttranscriptional pathways in utero and during lactation [29], that increase the susceptibility to develop metabolic disorders in offspring later in life [30, 31] has urge the need to better understand the mechanisms behind. In this study, we show that stressing dams with HFD during preconception, gestation and lactation has major effects on the developmental programming of AT in F1 offspring. These changes may determine the risk for developing metabolic complications later in life. While several studies have demonstrated the deleterious effect of moHF on offspring metabolism [7, 29, 32–34], the novelty of the present study is the combination of advanced physiological in vivo and molecular ex vivo techniques to further dissect the sex-dependent changes exerted by moHF on the offspring AT. Moreover, we highlighted complex transcriptional and posttranscriptional mechanisms by which these biological and physiological adaptations are sex- and adipose depot-dependent which may predispose differently female and male offspring to metabolic alterations later in life.

The embryo and fetus development are highly sensitive to its biological environments, and in late gestation, many fetal homeostatic adaptations can be readily perceived. BW of male offspring at weaning was altered by moHF, but not after 5-weeks post-weaning of HFD as opposed to offspring fed a CD [4, 6]. In the current study, the lipid load of the HF-fed offspring may counteract the lipid load received in utero from moHF. When the storage capacity of SAT is reduced, in high caloric overload, it leads to fat accumulation in other tissues including VAT and BAT, which promotes insulin resistance [35] and low-grade inflammation [36]. Males showed lower SAT and higher VAT accumulation (together with hyperplasia) than females at early-stage development, as well as whitening of the BAT, correlated with metabolic imbalance [8, 12]. At early stage, moHF impaired hepatic insulin sensitivity in females but this was normalized at END together with reduced inflammatory pathway activity in SAT, as also demonstrated in CD-fed female offspring [7, 37, 38].

Interestingly, the food intake was higher in F-moHF than F-moC with similar fat accumulation. This is likely compensated by an activation of BAT metabolism with increased *Ucp1, Pparα/γ*, and *Adrβ3* expression levels and TG remodeling in females, in line with others [39]. These results would support a protective mechanism in obese female offspring born from obese mothers, as opposed to males. The mechanisms by which moHF would modulate pathways in utero to prevent female, but not male, offspring from metabolic dysfunction later in life remains to be elucidated.

Both distribution and composition of the TG molecules into the adipocytes have consequences for metabolic risks. We show that there is a sex-dependent lipid profile in WAT at MID, but sex has a long-term effect (END) on the saturation level in SAT only, possibly due to readily available TG from the diet. Additionally, females redistribute TG profile in BAT. These major changes in AT lipid composition may be the result of the sex-dependent transcriptional activity between sexes as shown [13, 40]. Indeed, DEG analysis revealed that few genes were differently expressed between SAT and VAT in M-moHF, as opposed to females. This would indicate that males do not differentiate the transcriptional activity in the two white adipose depots which, in turn, will promote VAT growth and local inflammation.

Sex-dependent obesity-associated metabolic complications has been correlated to sex-dependent modulation of two major genes involved in adipogenesis (*Sfrp4*) [41] and in TG synthesis (*Dgat2*) [42]. In SAT, F-moHF promoted *Fabp4* (positive factor in FA signaling) and *Cited1* (X-chromosome-linked gene and a marker for beige cells) compared to M-moHF [26, 43]. In BAT, F-moHF repressed *Kng2* (blunt thermogenesis) [44] and induced *Elovl3* and *Mgll* (FA elongation and degradation), as opposed to M-moHF. Although the precise mechanism remains to be established, our results suggest that moHF reprograms AT toward more energy dissipation (Increased *Ucp1, Adrβ3* and decreased *Kng2*) and lipid oxidation (increased *Noct, Acad11* and *Ephx2*) in females. In males, reprogramming of AT led to an induction of WAT inflammation (induced *Ccl5/12/19* and *Cxcl12/15*), associated with impaired thermogenic function of BAT [45].

Moreover, another possible mechanism that could explain the sexual dimorphism is through the expression of X- and Y-linked specific genes. We have identified genes that escape the X-inactivation or have a Y paralog in offspring coming from both moC and moHF in the three metabolic tissues. Females showed higher expression of *Eif2s3x*, *Kdm6a*, *Kdm5c* and *Ddx3x* known as potential key regulators of adiposity [21, 46]. In male, Y-linked genes such as *Ddx3y* and *Eif2s3y* involved in cell-embryos development were upregulated in males, which would suggest a Y-chromosome signature that could contribute to the sexually dimorphism in offspring [47]. However, further research to define the role of X- and Y-linked genes in metabolism are necessary to elucidate the regulation of sexual dimorphic characteristics in response to moHF.

In conclusion, these novel findings may help to better prevent metabolic alterations in offspring and strongly support the concept that adipose depots have different metabolic functions due to different transcriptional regulation. In addition, we present further evidence that male and female offspring metabolic adaptation to moHF occurs in a sex- and adipose depot-dependent manner, which may set the basis for targeted medicine. These results provide unique information for the development of precision medicine for early identification of at-risk individuals according to the sex, and the development of novel therapeutic strategies to anticipate the development of obesity-related metabolic disorders.

## Materials and methods

More detailed description of the methods is included in the Supplementary Material.

### Mice and diet

All animal procedures were approved by the local Ethical Committee of the Swedish National Board of Animal Experiments. Four-week-old virgin dams and sire C57Bl6/J were ordered and recovered for one week before F0 dams were randomized to CD (moC; D12450H, Research Diets, NJ, USA; 10% kcal fat from soybean oil and lard; *n* = 6) or to HFD (moHF; D12451, Research Diets, NJ, USA; 45% kcal fat from soybean oil and lard; *n* = 6) for 6 weeks before mating. Sires remained on CD until mating. During this short mating period (up to 5 days) sires were on the same HFD as moHF dams (experimental unit). We assumed that the sires spermatozoa were unlikely affected by the HFD as sperm maturation time is ~35 days [48]. After mating, F0 sir and pregnant dams were separated. F0 dams were exposed to their respective diets until weaning. The F1 offspring were weaned at postnatal day 21 (3-week); male and female offspring were separated. Three to five animals were randomly housed per cage and fed with the HFD until the end of the study (Fig. 1a). The group of offspring born from HFD fed dams were named moHF and the group of offspring born from CD fed dams were named moC. All mice were housed in a 23 °C temperature-controlled 12 h light/dark room, with free access to water and food unless specified. The average food intake (per cage) in offspring was recorded twice a week for two weeks at around 4-month of age.

### In vivo magnetic resonance imaging and spectroscopy

The magnetic resonance imaging experiments were conducted on the same mouse at week 14 and week 25 using a 9.4 T horizontal bore magnet (Varian Yarnton UK) equipped with a 40 mm millipede coil, as previously described [49]. Localized ^1^H-MRS from visceral and subcutaneous fat depots were acquired from 2 × 1.5 × 1.5 mm^3^ voxels positioned in the upper gonadal abdominal fat (as representative of visceral fat) and in the inguinal abdominal fat (as representative of the subcutaneous fat) (Fig. 2a).

### Hematoxylin and eosin staining

Snap frozen AT samples were thawed in 4% paraformaldehyde and embedded, sectioned, and stained with hematoxylin-eosin according to standard procedures.

### Biochemical analysis of plasma

Glucose level was measured instantly via tail-nick one-touch glucometer. Extra blood was collected in a capillary tube for insulin measurement, using a commercial Insulin Elisa kit (EZRMI-13K).

### LC-MS analysis of triglycerides and gas chromatography fatty acid analysis

Total lipid extracts were separated using a high-performance liquid chromatography (HPLC) system (Ultimate 3000 Dionex, Thermo Fisher Scientific, Bremen, Germany). The identification of each TG species was validated by analysis of the MS/MS spectra. The MS/MS spectra of [M + NH_4_]^+^ ions of TGs allowed the assignment of the fatty acyl substituents on the glycerol backbone [50].

For fatty acids, total lipid extracts were obtained using a modified Bligh and Dyer method [51] and after transmethylation, the fatty acids were analyzed by gas chromatography with a flame ionization detector, adapted from [52].

### RNA isolation, qPCR, and sequencing

Total RNA was extracted from harvested tissues using QIAGEN miRNeasy Mini Kit (217004, Qiazol) and RNase-Free DNase Set (79254).

cDNA libraries were prepared for the bulk RNA sequencing in triplicates according to the Smart-Seq2 protocol [53]. Subsequently, the libraries were pooled into one lane for sequencing at a HiSeq3000 sequencer (Illumina), using sequencing format of dual indexing and single 50 base-pair reads.

For qPCR, cDNA was synthesized using a cDNA Synthesis Kit (product no. 1708891, Biorad) and was analyzed by qPCR using SYBR Green (SsoAdvanced™ Universal SYBR Green Supermix, product no. 172-5274, Biorad). The expression levels of the genes were normalized to housekeeping genes *β-Actin* and *Gapdh*.

### Bulk RNA-seq mapping and differential gene analysis

All raw sequence reads available in FastQ format was mapped to the mouse genome (mm10) using Tophat2 with Bowtie2 option [54, 55], where adapter sequences were removed using trim galore before read mapping. BAM files containing the alignment results were sorted according to the mapping position. Raw read counts for each gene were calculated using featureCounts from Subread package [56].

DEseq2 was used to perform the analysis of differential gene expression, where genes with raw counts as input [57]. The differentially expressed genes (DEG) were identified by adjust p value for multiple testing using Benjamini–Hochberg correction with false discovery rate values <0.1.

### Pathway analysis

The pathway analysis, also called Gene Set Enrichment Analysis [58], was performed using the KEGG pathways dataset.

### Statistical analyses

Differences between offspring sex and mother diet groups were determined using two-way ANOVA with diet (D) and sex (S) as independent variables, followed by Tukey’s multiple comparison post hoc test when significant (*p* < 0.05). Differences between two groups (sexes, F vs M; maternal diet moC vs moHF) were determined by *t*-test corrected for multiple comparisons using the Holm–Sidak method, with alpha = 5.000%. Samples size have been defined based on previous experiments. We assumed that we have sampled our data from a population that follows the Gaussian distribution.

## Supplementary information

Supplementary Method and Figures

## Data Availability

The raw data generated for RNA sequencing are available as described. For VAT and SAT SRA data: PRJNA662930 and BAT SRA data: PRJNA692177.
