# Supplementary material for: Maternal high-fat diet programs white and brown adipose tissue lipidome and transcriptome in offspring in a sex- and tissue-dependent manner in mice
Source: Int J Obes (Lond). 2022 Jan 7;46(4):831–42. doi: 10.1038/s41366-021-01060-5 (PMC8960419; doi:10.1038/s41366-021-01060-5)
Supplement: Supplementary file 1 — Supplementary Method and Figures [file 41366_2021_1060_MOESM1_ESM.pdf]

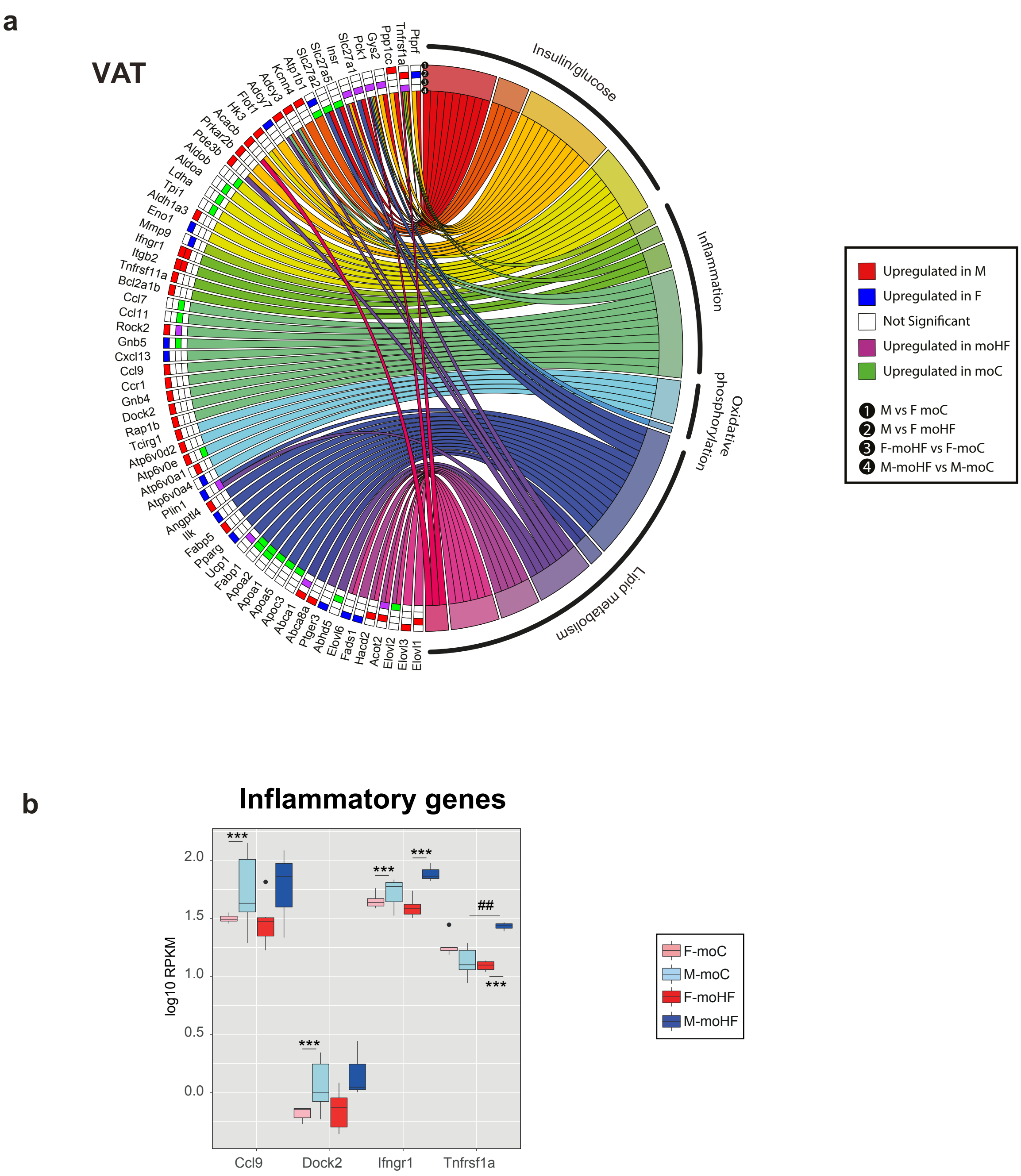

Supplementary Figure S1. Expression of genes in metabolic, inflammatory and oxidative pathways in VAT. a Chord plot displaying the DEG clustered into selected pathways. Female versus male (sex comparison) in (1) moC and (2) moHF, and moC versus moHF (maternal diet comparison)in (3) females and (4) males; **b** Boxplots presenting selected genes of the inflammatory pathway. F-moC, n=5, M-moC, n=5, F-moHF, n=6 and M-moHF, n=3. Benjamini-Hochberg correction with false Discovery Rate (FDR). values less than 0.1 when significant. *, M versus F and #, moHF versus moC, p < 0.05; ** or ##, p < 0.01; ***, p < 0.001.

a

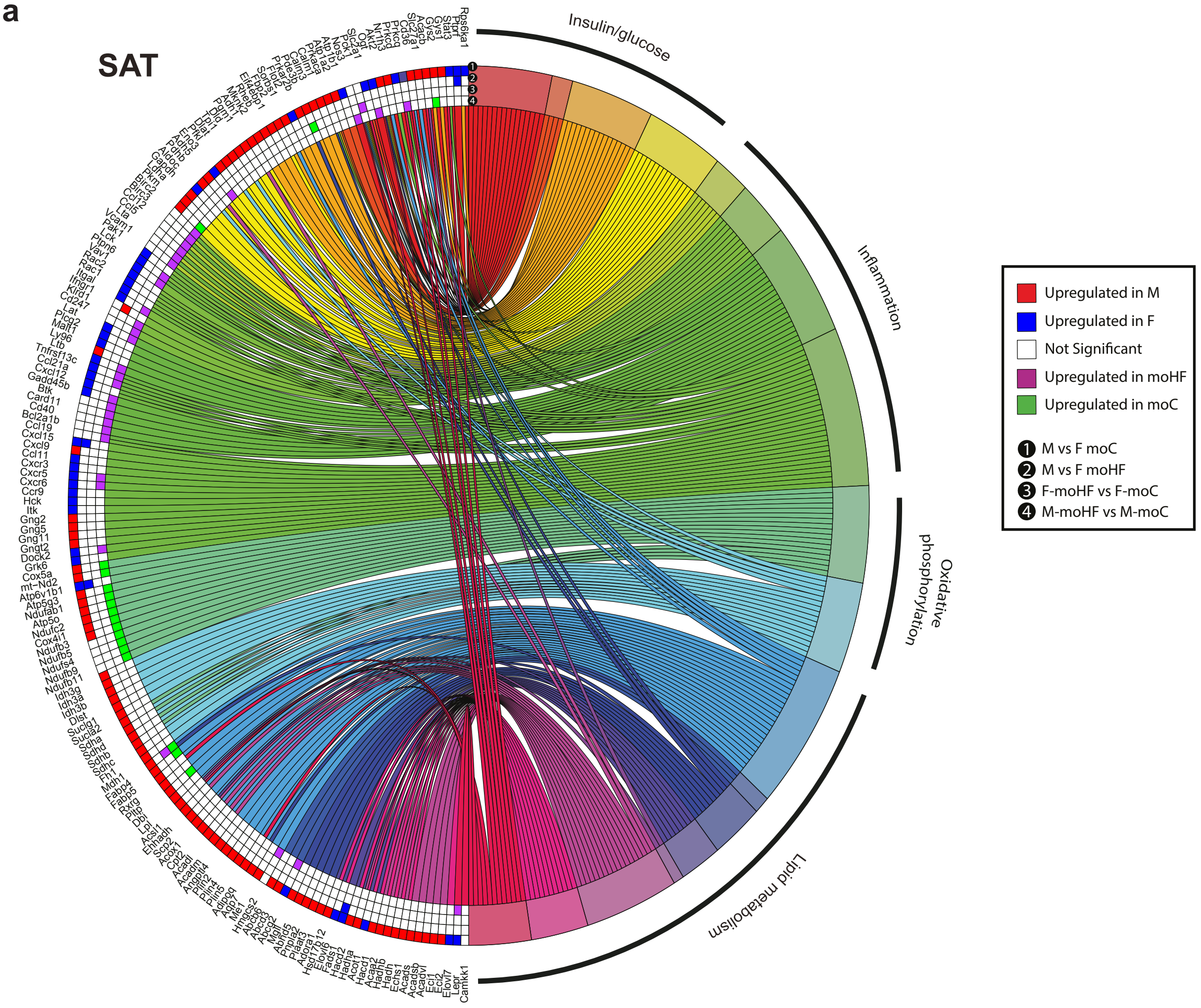

b

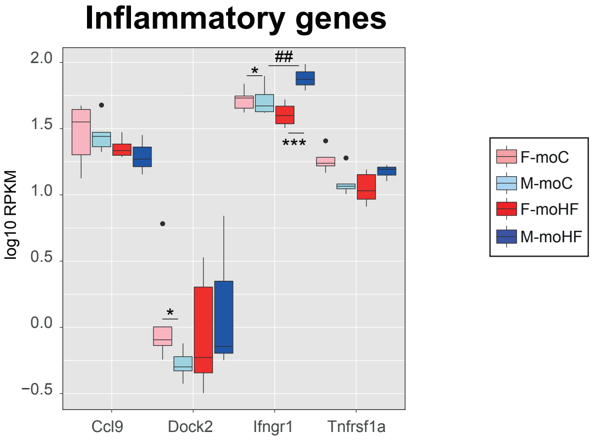

Supplementary Figure S2. Expression of genes in metabolic, inflammatory and oxidative pathways in SAT. **a** Chord plot displaying the DEG clustered into selected pathways. Female versus male (sex comparison) in (1) moC and (2) moHF, and moC versus moHF (maternal diet comparison) in (3) females and (4) males; **b** Boxplots presenting genes of the inflammatory pathway. F-moC, n=5, M-moC, n=5, F-moHF, n=6 and M-moHF, n=3. Benjamini-Hochberg correction with false Discovery Rate (FDR). values less than 0.1 when significant. *, M versus F and #, moHF versus moC, $p < 0.05$; ** or ##, $p < 0.01$; ***, $p < 0.001$.

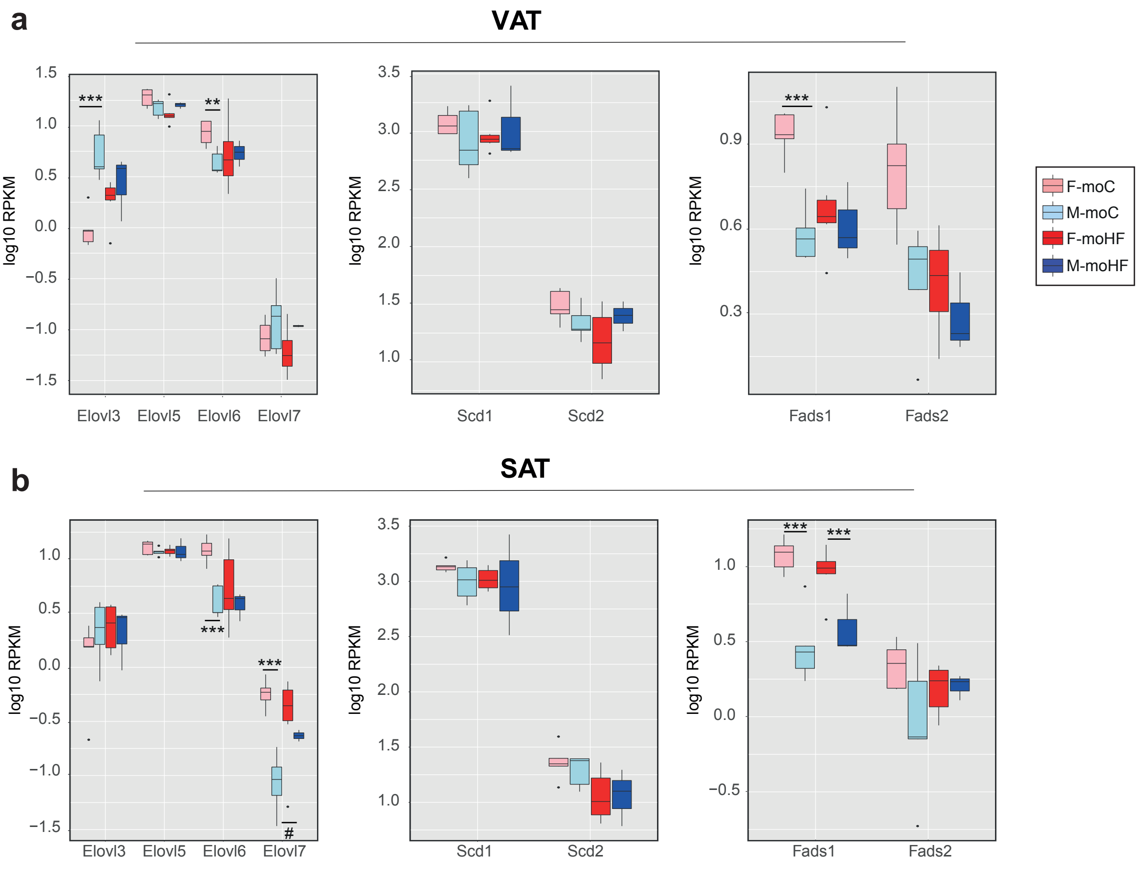

Supplementary Figure S3. Gene expression levels from the lipid elongation and desaturation pathways. Boxplots presenting genes of the lipid synthesis pathway in **a** VAT and **b** SAT. F-moC, n=5, M-moC, n=5, F-moHF, n=6 and M-moHF, n=3. Benjamini-Hochberg correction with false Discovery Rate (FDR) values less than 0.1 when significant. *, M versus F and #, moHF versus moC, $p < 0.05$; ** or ##, $p < 0.01$; *** or ###, $p < 0.001$.

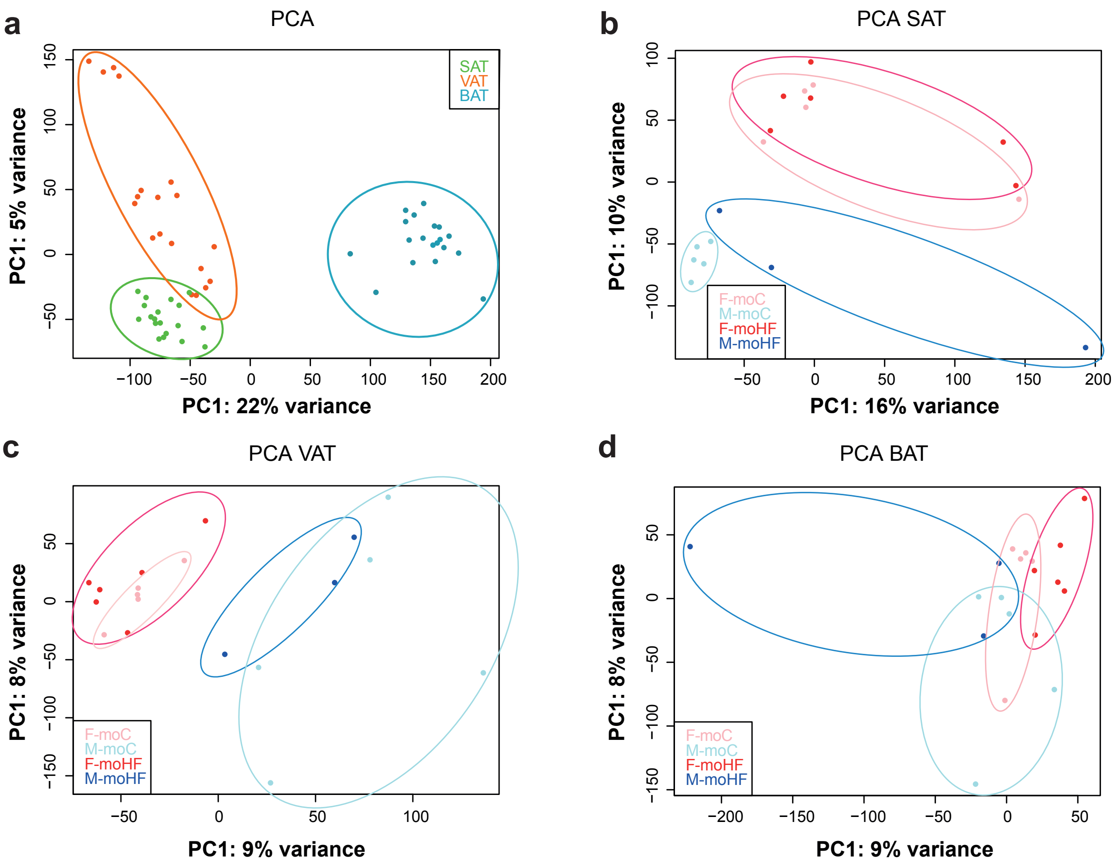

Supplementary Figure S4. Principal component analysis (PCA) plots from RNA sequencing dataset. a PCA plot of all samples from VAT, SAT and BAT offspring. The orange color indicates the samples from VAT, the green from SAT and the blue from BAT. PCA plot of **b** SAT, **c** VAT and **d** BAT dataset of females and males from moC and moHF. F-moC, n=5, M-moC, n=5, F-moHF, n=6 and M-moHF, n=3. The light pink color indicates the samples from F-moC, light blue indicates the M-moC, the dark pink indicates the F-moHF and the dark blue indicates the M-moHF samples.

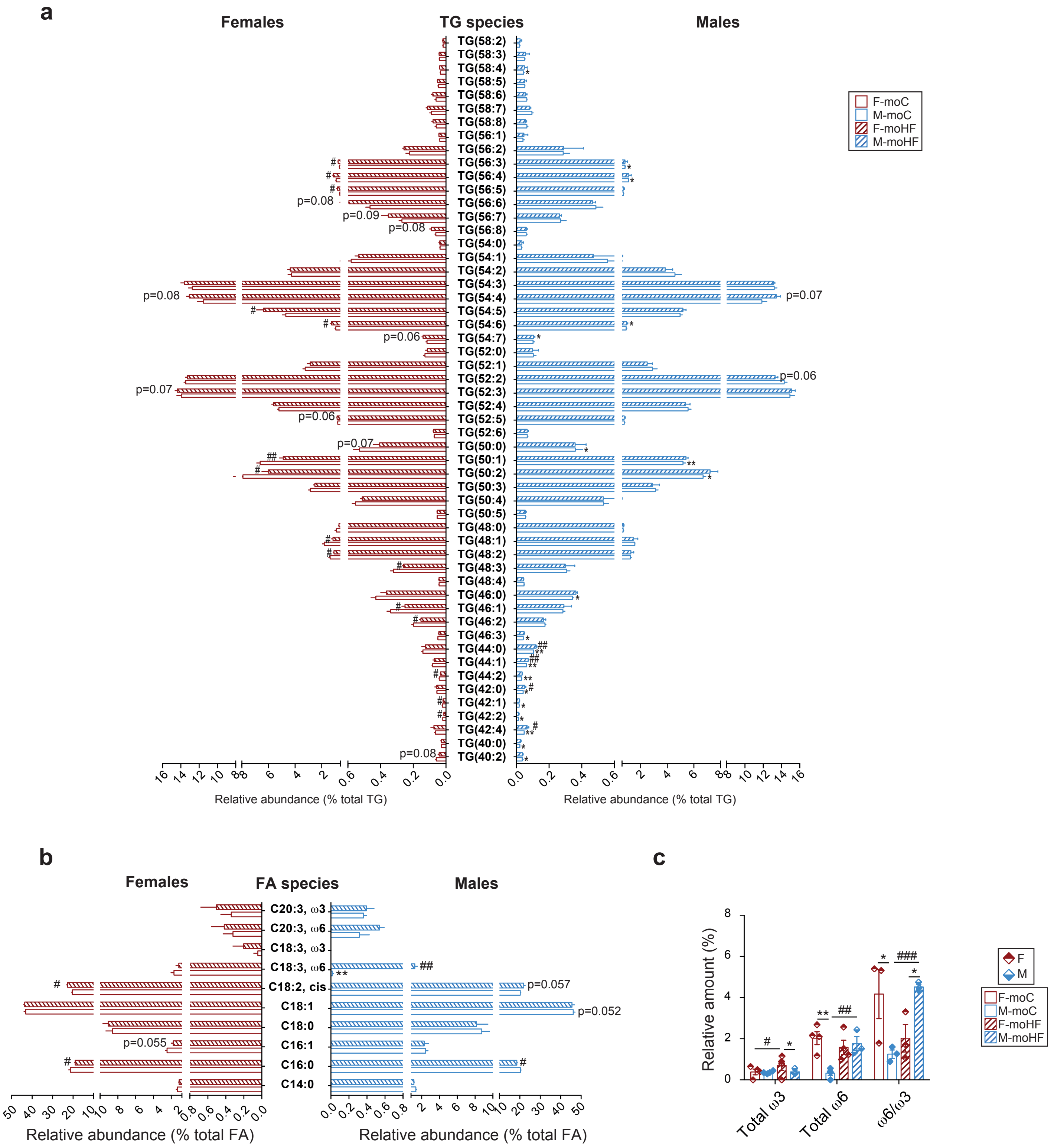

Supplementary Figure S5. TG and FA species extracted from BAT.
a Plot of all TG species detected by LC-MS in females and males offspring from moC (n=4) and moHF (n=4); **b** Plot of the FA species detected by GC-MS (n=4) in the TG; **c** The ω–3 and ω–6 FA pathways and the ratio of ω–6 to ω–3 FA pathways. Differences between two groups (sexes, F versus M; maternal diet, moC versus moHF) were determined by unpaired t-test corrected for multiple comparisons using the Holm–Sidak method, with alpha = 5.000%. *, M versus F and #, moHF versus moC, $p < 0.05$; ** or ##, $p < 0.01$; *** or ###, $p < 0.001$.

MATERIALS AND METHODS

In vivo magnetic resonance imaging and spectroscopy

Animals were anesthetized using isoflurane (4% for sleep induction and ~2% for sleep maintenance) in a 3:7 mixture of oxygen and air, before being positioned prone in the MR-compatible animal holder. Respiration was monitored during scanning (SA-instruments, Stony Brook, NY, USA). Core body temperature was maintained at 37°C during scanning using a warm air system (SA-instruments, Stony Brook, NY, USA). For magnetic resonance spectroscopy, heart beats were recorded using an electrocardiogram system as previously described (1). Fiji software (http://fiji.sc) was used to compute the volume of total fat (TF), visceral adipose tissue (VAT), subcutaneous adipose tissue (SAT) and brown adipose tissue (BAT).

In vivo localized proton magnetic resonance spectra (^{1}H-MRS)

Point Resolved Spectroscopy (PRESS) was used as primary pule sequence (2) with the following parameters: time to echo 15 ms, sweep width 8013 Hz, number of excitations 16, refocusing pulses 1.6 ms mao pulses with a band width nominal bandwidth of 2936 Hz as described (1). All spectroscopy data were processed using the LCModel analysis software (http://s-provencher.com/pub/LCModel/manual/manual.pdf). “Lipid 6” for adipose spectrum were used as a base with all signals occurring in the spectral range of 0 to 7 ppm (water resonance at 4.7ppm) simulated in LCModel. All concentrations were derived from the area of the resonance peaks of the individual metabolites. Only the fitting results with an estimated standard deviation of less than 20% were further analyzed. ^{1}H-MRS spectra revealed nine lipid signals (peaks) in the mouse adipose, based on published data (2). As for the MRI, ^{1}H-MRS experiments were repeated twice on the same animal at MID and END.

The properties of triacylglycerols were calculated from the area under the curve (AUC_1–AUC_9) of the nine lipid peaks (L1–L9) according to published work (3).

The mean chain length (MCL)

$$MCL = \frac{\frac{1}{3} * L1 + \frac{1}{2} * (L2 + L3 + L4 + L5 + L6) + L9}{\frac{1}{3} * L1} + 1$$

Where it is assumed that all saturated fatty acids have a chain length of 16 and the polyunsaturated fatty acids a chain length of 18, as other fatty acids are rare and therefore will change only slightly the value of the MCL (4).

The fraction of unsaturated fatty acids (fUL)

$$fUL = \frac{3}{4} * \frac{L4}{L1}$$

The fraction of saturated fatty acids (fSL)

$$fSL = 1 - fUL$$

The fraction of polyunsaturated fatty acids (fPUL)

$$fPUL = \frac{3}{2} * \frac{L6}{L1}$$

And the fraction of monounsaturated (fMUL)

$$fMUL = fUL - fPUL$$

Hematoxylin and eosin (H&E) staining of adipose tissue

Adipocyte hyperplasia and hypertrophy were analyzed automatically using Adiposoft version 1.16 (https://imagej.net/plugins/adiposoft) plugin software program for ImageJ,

version 2.1.0/1.53c (https://imagej.nih.gov/ij/index.html). 0.251 microns per pixel with a minimum diameter of 20 and a maximum of 150 were used. The area output unit was μm^2. Five random areas of the section were examined in 40x magnification for hyperplasia and hypertrophy and analyzed automatically.

Biochemical analysis of plasma

Based on glucose and insulin levels Matsuda index (whole body insulin sensitivity index) and direct measurement of hepatic insulin resistance (HOMA index) were calculated as described (5, 6). Briefly, homeostatic model assessment (HOMA) index was calculated as follows = (I_0 x G_0)/22.5. Matsuda index was calculated as = $10000/(\sqrt{[G_0 \times I_0 \times G_{mean} \times I_{mean}]})$, the suffix *mean* indicates the average value of glucose and insulin levels measured during the whole length of the glucose test. Evaluation of β-cell function was calculated by dividing the area under the curve (AUC) of insulin and glucose levels during the glucose test (AUCins:AUCglc).

LC-MS analysis of triglycerides

HPLC system with an autosampler coupled online to a Q-Exactive hybrid quadrupole Orbitrap mass spectrometer (Thermo Fisher Scientific, Bremen, Germany), adapted from (7, 8) was used for total lipid separation. LC-MS analysis was carried out using an Accucore™ C30 column (150 × 2.1 mm) that was equipped with 2.6 µm diameter fused-core particles (Thermo Fisher Scientific, Germering, Germany). The solvent system consisted of two mobile phases: mobile phase A (water/ACN 50/50 (v/v) with 0.1% formic acid and 5 mM ammonium formate) and mobile phase B (isopropanol/ACN/water 85/10/5 (v/v) with 0.1% formic acid and 5 mM ammonium formate). Initially, 50% of mobile phase B was held isocratically for 2 min, followed by a linear increase to 86% of B within 18 min. An increase to 95% B occurred in 1 min, which was held up for 14 min, returning to the initial conditions (50% B) in 2 min, followed by a re-equilibration period of 8 min prior to the next injection. An aliquot of 20 µg of each lipid extract

were dissolved in 80 µL of MeOH. Two µL of each dilution were introduced into the Accucore™ C30 column (150 × 2.1 mm) that was equipped with 2.6 µm diameter fused-core particles (Thermo Fisher Scientific, Germering, Germany) with a flow rate of 300 µL min^{-1}. The temperature of the column oven was maintained at 40°C. The mass spectrometer with Orbitrap technology operated in positive (electrospray voltage 3.0 kV) ion mode with a capillary temperature of 350°C, a sheath gas flow of 45 arbitrary units (a.u), an auxiliary gas flows of 15 a.u., a high resolution of 70 000, a maximum injection time of 100 ms and AGC target 1e6. In MS-MS experiments, cycles consisted of one full scan mass spectrum and ten data-dependent MS-MS scans (resolution of 17 500, a maximum injection time of 100 ms an AGC target of 1e5, and with an isolation window of 1 *m/z*). Cycles were repeated continuously throughout the experiments with the dynamic exclusion of 60 s and an intensity threshold of 5e4. Normalized collisional energy ranged between 20, 23, and 25 eV. C30 RP-LC-MS spectra from molecular species of triglyceride were analyzed in positive ion mode and TG were identified as $[M+NH_4]^+$ ions. Data acquisition was carried out using the Xcalibur data system (V3.3, Thermo Fisher Scientific, USA). The mass spectra were processed and integrated through the MZmine software (v2.32) (9). This software allows for filtering and smoothing, peak detection, alignment and integration, and assignment against an in-house database, which contains information on the exact mass and retention time for each TG molecular species. During the processing of the data by MZmine, only the peaks with raw intensity higher than 1e4 and within 5 ppm deviation from the lipid exact mass were considered.

Fatty acid analysis using gas chromatography with a flame ionization detector (GC-FID)

Fatty acid methyl esters (FAMEs) were dissolved in 30 µL of *n*-hexane and 2.0 µL were injected in GC-FID (PerkinElmer Clarus 400 gas chromatograph (Waltham, MA). The gas chromatograph injection port was programmed at 215°C and the detector at 250°C. The initial temperature was 75°C and the oven temperature was programmed in 3 ramps (a 15°C/min increase to 163°C for 2 min, a 2°C/min increase to 175°C for 2 min, and a 10°C/min increase

to 250°C form 5 min), performed for 28.3 min in total. Hydrogen was the carrier gas (flow rate, 1.7 ml/min). A DB-FFAP column (30m long, 0.32 mm internal diameter, and 0.25 μm film thickness (J & W Scientific, Folsom, CA, USA)) was used. Peaks corresponding to each FA were identified based on retention time in comparison with a Supelco 37 Component FAME standard mixture (Sigma-Aldrich, USA), integrated and the percentage of each FA was related to the sum area of all FAs identified. The total ω-3 content was calculated as the summed total of ω-3 PUFA of C18:3ω-3 and C20:3ω-3. Total ω-6 content was calculated as the summed total of C18:2ω-6 and C20:3ω-6 contents.

RNA isolation, purity and sequencing

RNA concentration was measured by nanodrop and diluted to 2.17 ng/μl. RNA quality was assessed for 12 out of 57 samples randomly by a 2100 Bioanalyzer using a nano chip (Agilent Biotechnologies), and RIN numbers were between 8.7-9.9. cDNA libraries were prepared for the bulk-RNA sequencing in 384-well plate with triplicates for each RNA sample according to the previously described Smart-Seq2 protocol (10). In brief, mRNA was transcribed into cDNA using oligo(dT) primer and SuperScript II reverse transcriptase (ThermoFisher Scientific). Second strand cDNA was synthetized using a template switching oligo, followed by PCR amplification for 15 cycles. Purified cDNA was quality controlled on a 2100 Bioanalyzer with a DNA High Sensitivity chip (Agilent Biotechnologies), fragmented and tagged (tagmented) using Tn5 transposase, and each single sample was uniquely indexed using the Illumina Nextera XT index kits (Set A-D).

Pathway analysis

First, genes were ranked descending according to the Log2 Fold Change (Log2FC) of expression. For each query pathway, if gene *i* is a member of the pathway, it is defined as

$$Xi = \sqrt[2]{\frac{N-G}{G}}$$

If gene *i* is not a member of the pathway, it is defined as

$$X_i = -\sqrt[2]{\frac{G}{N-G}}$$

where *N* indicates the total number of genes and *G* indicates the number of genes in the query pathway. Next, a max running sum across all *N* genes Maximum Estimate Score (MES) is calculated as

$$MES = \max_{1 \leq j \leq N} \sum_{i=1}^{j} Xi$$

The permutation test was performed with 1000 times to judge the significance of MES values. The query pathway with a nominal p-value less than 0.05 and FDR values less than 0.1 would be considered to be significantly enriched. The positive MES value indicates up-enrichment (up-regulation) whereas a negative MES value indicates down-enrichment (down-regulation) of a pathway.

Unsupervised clustering.

The raw data matrix was distributed column-wise by sample IDs and row-wise by gene names. The TMM method was used to normalize between samples (11). Unsupervised clustering was then performed using the Principal Component Analysis (PCA) plot function in R. The PCA plot is based on the two most variant dimensions in which the gene parameters with duplicated data are filtered out.

REFERENCES

1. Korach-Andre M. In Vivo Investigation of High-Fat Diet-Induced Hepatic Lipid Dysfunctions. Methods Mol Biol. 2020;2164:109-19.
2. Strobel K, van den Hoff J, Pietzsch J. Localized proton magnetic resonance spectroscopy of lipids in adipose tissue at high spatial resolution in mice in vivo. J Lipid Res. 2008;49(2):473-80.
3. Ye Q, Danzer CF, Fuchs A, Vats D, Wolfrum C, Rudin M. Longitudinal evaluation of hepatic lipid deposition and composition in ob/ob and ob/+ control mice. NMR Biomed. 2013;26(9):1079-88.

4. Raclot T, Groscolas R. Selective mobilization of adipose tissue fatty acids during energy depletion in the rat. J Lipid Res. 1995;36(10):2164-73.
5. Matsuda M, DeFronzo RA. Insulin sensitivity indices obtained from oral glucose tolerance testing: comparison with the euglycemic insulin clamp. Diabetes Care. 1999;22(9):1462-70.
6. Pacini G, Omar B, Ahren B. Methods and models for metabolic assessment in mice. J Diabetes Res. 2013;2013:986906.
7. Anjos S, Feiteira E, Cerveira F, Melo T, Reboredo A, Colombo S, et al. Lipidomics Reveals Similar Changes in Serum Phospholipid Signatures of Overweight and Obese Pediatric Subjects. J Proteome Res. 2019;18(8):3174-83.
8. Colombo S, Melo T, Martinez-Lopez M, Carrasco MJ, Domingues MR, Perez-Sala D, et al. Phospholipidome of endothelial cells shows a different adaptation response upon oxidative, glycative and lipoxidative stress. Sci Rep. 2018;8(1):12365.
9. Pluskal T, Castillo S, Villar-Briones A, Oresic M. MZmine 2: modular framework for processing, visualizing, and analyzing mass spectrometry-based molecular profile data. BMC Bioinformatics. 2010;11:395.
10. Picelli S, Faridani OR, Bjorklund AK, Winberg G, Sagasser S, Sandberg R. Full-length RNA-seq from single cells using Smart-seq2. Nat Protoc. 2014;9(1):171-81.
11. Robinson MD, Oshlack A. A scaling normalization method for differential expression analysis of RNA-seq data. Genome Biol. 2010;11(3):R25.
